# A Decade of Molecular Understanding of Withanolide Biosynthesis and *In vitro* Studies in *Withania somnifera* (L.) Dunal: Prospects and Perspectives for Pathway Engineering

**DOI:** 10.3389/fpls.2015.01031

**Published:** 2015-11-27

**Authors:** Niha Dhar, Sumeer Razdan, Satiander Rana, Wajid W. Bhat, Ram Vishwakarma, Surrinder K. Lattoo

**Affiliations:** ^1^Plant Biotechnology, CSIR – Indian Institute of Integrative MedicineJammu Tawi, India; ^2^Medicinal Chemistry, CSIR – Indian Institute of Integrative MedicineJammu Tawi, India

**Keywords:** *Withania somnifera*, withanolides, tissue culture, elicitor, medicinal plant, molecular cloning, secondary metabolites, pathway engineering

## Abstract

*Withania somnifera*, a multipurpose medicinal plant is a rich reservoir of pharmaceutically active triterpenoids that are steroidal lactones known as withanolides. Though the plant has been well-characterized in terms of phytochemical profiles as well as pharmaceutical activities, limited attempts have been made to decipher the biosynthetic route and identification of key regulatory genes involved in withanolide biosynthesis. This scenario limits biotechnological interventions for enhanced production of bioactive compounds. Nevertheless, recent emergent trends vis-à-vis, the exploration of genomic, transcriptomic, proteomic, metabolomics, and *in vitro* studies have opened new vistas regarding pathway engineering of withanolide production. During recent years, various strategic pathway genes have been characterized with significant amount of regulatory studies which allude toward development of molecular circuitries for production of key intermediates or end products in heterologous hosts. Another pivotal aspect covering redirection of metabolic flux for channelizing the precursor pool toward enhanced withanolide production has also been attained by deciphering decisive branch point(s) as robust targets for pathway modulation. With these perspectives, the current review provides a detailed overview of various studies undertaken by the authors and collated literature related to molecular and *in vitro* approaches employed in *W. somnifera* for understanding various molecular network interactions in entirety.

## Introduction

Plants have long been known to allocate substantial resources toward developing chemical solutions to enhance survival strategies in the form of varied natural products. These natural products have been scrupulously used as pharmaceuticals, additives, pesticides, agrochemicals, fragrance and flavor ingredients, food additives, and pesticides. A great majority of these plant derived natural products have long been the basis of many traditional medicines and still they continue to provide mankind with new remedies. Since time immemorial medicinal plants and their extracts have been used by humans for the treatment of different ailments and diseases. For instance, oils of *Cedrus* species (Cedar), *Cupressus sempervirens* (Cypress), *Glycyrrhiza glabra* (Licorice), *Commiphora* species (Myrrh), and *Papaver somniferum* (Poppy juice), all of which are still in use today for the treatment of ailments ranging from coughs and colds to parasitic infections and inflammation. Many of the modern drugs against various ailments are also based on the chemical structures of such plant derived chemical products. During the period of 2005–2007 the Food and Drug Administration introduced 13 new drugs of natural origin into the market and more than 100 natural product-based drugs are in clinical studies (Li and Vederas, 2011). Today, all drugs used in western medicine, around 40–45% are natural products or compounds derived from them, and of these, 25% are obtained from plants. Moreover, the dominant role of natural products like vinca alkaloid derivatives (etoposide, teniposide, etoposide phosphate) acting against cancer (60%) and infectious diseases (75%) is all the more identified (Mander and Liu, 2010). In recent years, from a health perspective, protective dietary constituents in the form of plant derived natural compounds have become progressively significant part of human nutrition research (Pandey and Rizvi, 2009; Choi et al., 2012).

Such vast range of chemical entities in the form of natural products that do not contribute directly in growth and development of a plant are termed as secondary metabolites. Plant secondary metabolites like phenylpropanoids, terpenoids, and alkaloids play significant role in plant survival under specialized ecological conditions, e.g., biotic and abiotic stresses. In contrast with primary metabolites, secondary metabolites are often restricted in distribution and in many instances a specific secondary metabolite is associated with a specific taxonomic groups or a plant species. Medicinal plants have long been the basis of herbal drugs for prevention and treatment of various ailments and secondary metabolites are attributed with the medicinal properties (Croteau et al., 2000; Rao and Ravishankar, 2002). These herbal drugs have thus been in use for thousands of years in different cultures due to their potency, efficacy, low cost, and fewer side-effects. With an ever-increasing global demand for herbal medicine, there is not only requirement for large quantity of raw material of medicinal plants, but also of appropriate quality where active principles are available in desired concentrations (Shahid et al., 2013). Additionally, due to the complex chemical structures, it is often difficult to synthesize complex natural compounds through synthetic chemistry as the whole process is economically prohibitive. Thus, plants remain as a sole sustainable natural resource of many medicinally important secondary metabolites.

The biosynthesis of plant secondary metabolites is tightly regulated by spatial and temporal cues that limit the levels of targeted secondary metabolites in plant tissues (Dhar et al., 2013). Furthermore, many secondary metabolites are often species specific in distribution and the plant species in question may be distributed to specific geographical location. Cumulatively, these issues may limit proper exploitation of plants for large scale production of economically important secondary metabolites. For obvious reasons, a desirable aspect is to improve the level of secondary metabolites in native plant species as well as to develop alternative technologies to produce high value bioactive compounds in microbial or yeast heterologous hosts by using synthetic biology approaches. In this regard, molecular biotechnological interventions and *in vitro* approaches offer attractive possibilities for metabolic engineering of plant secondary metabolites. However, biogenesis of several important plant secondary metabolites at the level of pathway steps and their regulation is poorly understood. These issues can be attributed to the lack of functional genomics platforms comprising of genome resources, mutants, and transformation systems for medicinal plants. Hence, refinement in tools and techniques to carry out comprehensive studies of medicinal plant secondary metabolism is imperative.

Herein, we provide a comprehensive review of the detailed studies carried out by the authors and other significant contributions collated from the available literature to elucidate different aspects of biosynthesis of steroidal lactone compounds known as withanolides from *W. somnifera*, a medicinal plant of immense repute. It also entails many important aspects related to enhancement of withanolide production in corroboration with molecular deciphering and *in vitro* approaches to understand the regulation of withanolide production.

## Withania somnifera

*Withania somnifera* (Solanaceae) commonly known as ashwagandha or Indian ginseng, is a valued medicinal plant known since antiquity (~3000 years; Winters, 2006). It is a commended genus described in the Indian Ayurvedic system of medicine and also enlisted as an important herb in Unani and Chinese traditional medicinal systems. It displays an efficient reproductive behavior of mixed mating which enables it to maximize benefits of both selfing as well as outcrossing. Its breeding behavior also guarantees wide chemotypic variability (Lattoo et al., 2007). *W. somnifera* is widely distributed around the world and is mainly adapted to xeric and drier regions of tropical and subtropical domains, ranging from the Canary Islands, the Mediterranean region and Northern Africa to Southwest Asia (Mirjalili et al., 2009).

Traditionally, *W. somnifera* is recommended to enhance physiological endurance, overall vitality, strength and general health. It is frequently equated with Korean ginseng (*Panax ginseng*) for its restorative bioactivities. It also aids to offset impotency, chronic fatigue, weakness, bone weakness, dehydration, premature aging, muscle tension, and emaciation. All parts of the plant, like leaves, stem, flower, root, seeds, and bark are used medicinally. Root of *Withania* is an important ingredient of more than 200 formulations in traditional systems of medicine like Ayurveda, Siddha, and Unani. These systems are being used for ages in the management of numerous physiological ailments (Sukanya et al., 2010). The bitter leaves of the plant have characteristic odor, used as an antihelmantic and infusion is given in fever. In Ayurveda, berries and tender leaves are prescribed to be applied externally to tumors, tubercular glands, carbuncles, and ulcers (Gauttam and Kalia, 2013).

## Withanolides

**Figure 1:**
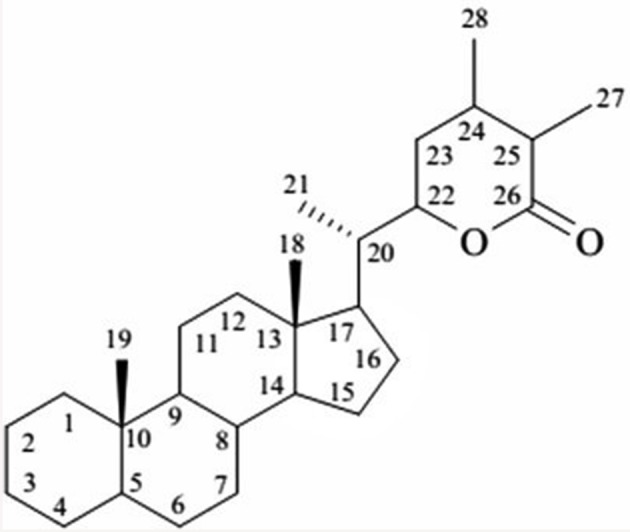
**The basic structure of withanolide**.

*W. somnifera* is known to structure a wide-range of low molecular weight secondary metabolites for example terpenoids, flavonoids, tannins, alkaloids, and resins. It has been extensively studied for its chemical constituents that include compounds of diverse chemical structures viz. withanolides, alkaloids, flavonoids, tannin (Elsakka et al., 1990; Attaurrahman et al., 1991; Arshad Jamal et al., 1995; Choudhary et al., 2010). Of these, withanolides are credited with widely acclaimed remedying properties. Withanolides, with as many as 40 reported structures represent a collection of naturally occurring C-28 steroidal lactone triterpenoids assembled on an integral or reorganized ergostane structure, in which C-22 and C-26 are oxidized to form a six-membered lactone ring (Glotter, 1991; Ray and Gupta, 1994). The elementary structure is labeled as the withanolide skeleton chemically nomenclatured as 22-hydroxy ergostane-26-oic acid 26, 22-lactones (Misra et al., 2008). The withanolides are generally polyoxygenated and believed to be produced *via* enzyme system capable of catalyzing oxidation of all carbon atoms in a steroid nucleus (Kirson et al., 1971; Figure 1).

The characteristic feature of withanolides and ergostane-type steroids is one C-8 or C-9 side chain with a either six or five membered lactone or lactol ring. This lactone ring could be attached through a carbon-carbon bond or through an oxygen bridge with the carbocyclic part of the molecule (Kirson et al., 1971; Glotter, 1991). Various kinds of structural rearrangements involving oxygen substituents like bond scission, new bond formation, ring aromatization among others can result in formation of novel structural variants often described as modified withanolides or ergostane type steroids (Misico et al., 2011). They are distributed in distinct amounts and ratios in fruits and vegetative parts of the plant (Sangwan et al., 2008). However, withanolides are mainly localized to leaves, and their concentration is generally low i.e., ranges from 0.001 to 0.5% of dry weight basis (DWB). Many factors such as growth rate, geographical, and environmental conditions are known to modulate the content of withanolides (Dhar et al., 2013). Apart from *Withania*, withanolides are also distributed to other Solanaceous genera like *Iochroma, Acnistus, Deprea, Datura, Lycium, Dunalis, Nicandra, Jaborosa, Physalis, Salpichroa, Tubocapsicum, Discopodium, Trechonaetes*, and *Witheringia*. Moreover, their distribution is not restricted completely to Solanaceous plants, withanolides have been reported to be isolated from Taccaceae, Leguminosae (Glotter, 1991), Dioscoreaceae (Kim et al., 2011), Myrtaceae, and Lamiaceae families including reports of isolation from marine organisms also (Chao et al., 2011).

## Pharmacology

During last two decades, there has been a notable surge in the pharmacological based research of this plant as evidence for anti-tumor, anti-arthritic, anti-aging, and neuroprotective properties (Budhiraja and Sudhir, 1987; Ray and Gupta, 1994; Jayaprakasam et al., 2003; Choudhary et al., 2005; Kuboyama et al., 2005; Kaileh et al., 2007; Singh et al., 2011) and also positive influences on the endocrine, cardiopulmonary, and central nervous systems have been reported (Mishra et al., 2000). Anabalagan and Sadique (1981) reported efficient anti-inflammatory activity of *W. somnifera* as compared to hydrocortisone. *W. somnifera* extracts have revealed anti-inflammatory effects in a range of rheumatological conditions (al-Hindawi et al., 1992). *W. somnifera* preparations are reported to influence the cholinergic and GABA-ergic neurotransmission (GABA: γ-amino-butyric acid) accounting for various central nervous system related disorders (Kulkarni and George, 1996; Tohda et al., 2005). The active principles, sitoindosides VII–X and withaferin A (WS-3) significantly reduces the lipid peroxidation and increased levels of the superoxide dismutase, catalase, glutathione peroxidase, and ascorbic acid activity, thus possessing a free radical scavenging property (Bhattacharya et al., 1997; Panda and Kar, 1997). Further, normal and cyclophosphamide-treated mice showed enhanced levels of interferon-γ, interleukin-2, and granulocyte macrophage colony stimulating factor by exhibiting immuno-potentiating and myeloprotective effects (Davis and Kuttan, 1999). Further, toxicity studies have shown withanolides to be safer compounds with minute or no related toxicity (Mishra et al., 2000). Withanolides have been noticed to enhance the ability of macrophage to “eat” pathogens (Davis and Kuttan, 2000). WS-3 acts defending in definite types of cancers as an inhibitor of angiogenesis (Mohan et al., 2004). Other studies reveal that withanolides possess potent antimicrobial potential against pathogenic bacteria, including *Salmonella*, an organism associated with food poisoning (Owais et al., 2005). Withanoside VI and withanolide A (WS-1) facilitate the regeneration of axons and dendrites by reconstructing pre- and post-synapses in neurodegenerative diseases and preventing pathogenesis and neuronal death. It is found to ameliorate the memory deficit in mice (Kuboyama et al., 2005, 2006). Mechanistically, withanolides act as anti-inflammatory agents by inhibiting lymphocyte proliferation, complement system, land delayed-type hypersensitivity (Rasool and Varalakshmi, 2006). WS-3 is a promising agent for the treatment of the inflammatory cascade of cardiovascular diseases as a potent inhibitor of the pro-inflammatory transcription factors (Kaileh et al., 2007). It exhibits *in vivo* anti-cancer activity against pancreatic cancer by inhibiting Hsp90 chaperone activity and another potential withanolide isolated from roots act as an effective agent to protect against skin carcinoma induced by UV-B (Mathur et al., 2004; Yu et al., 2010). Withanolidesulfoxide have been isolated and identified to inhibit COX-2 enzyme in various tumor cell lines and to suppress their proliferation (Mulabagal et al., 2009). Withanolide D can be used with traditional chemotherapeutic agents as it augments the ceramide accretion by triggering neutral-sphingomyelinase 2, modulate phosphorylation of the JNK and p38MAPK and induced apoptosis in both myeloid and lymphoid cells along with primary cells derived from leukemia patients (Mondal et al., 2010). WS-3 and withanolide D have been demonstrated to hamper angiogenesis, Notch-1, NFκB in cancer cells and trigger apoptosis in breast cancer cells (Kaileh et al., 2007; Koduru et al., 2010; Hahm et al., 2011). The oral administration of withanolides and withanosides reversed behavioral deficits, plaque pathology, accumulation of β-amyloid peptides (Aβ) in the animal models of Alzheimer disease through higher expression of low-density lipoprotein receptor-related protein in brain micro-vessels and the Aβ-degrading protease neprilysin (Sehgal et al., 2012). Recently, WS-3 was found to activate Cdc2 protein in prostate cancer cell lines which result in arrest of the cell cycle and leads to cell death (Roy et al., 2013) and the root extract have been observed to possess neuroprotective effect against β-amyloid and HIV-1Ba-L (clade B) induced neuro-pathogenesis (Kurapati et al., 2013). The other withanolides and alkaloids like withasomine, cuseohygrine, and anahygrine remain to be the promising lead-compounds for the development of the new anti-inflammatory drugs (Mirjalili et al., 2009).

## Biosynthesis of withanolides

**Figure 2:**
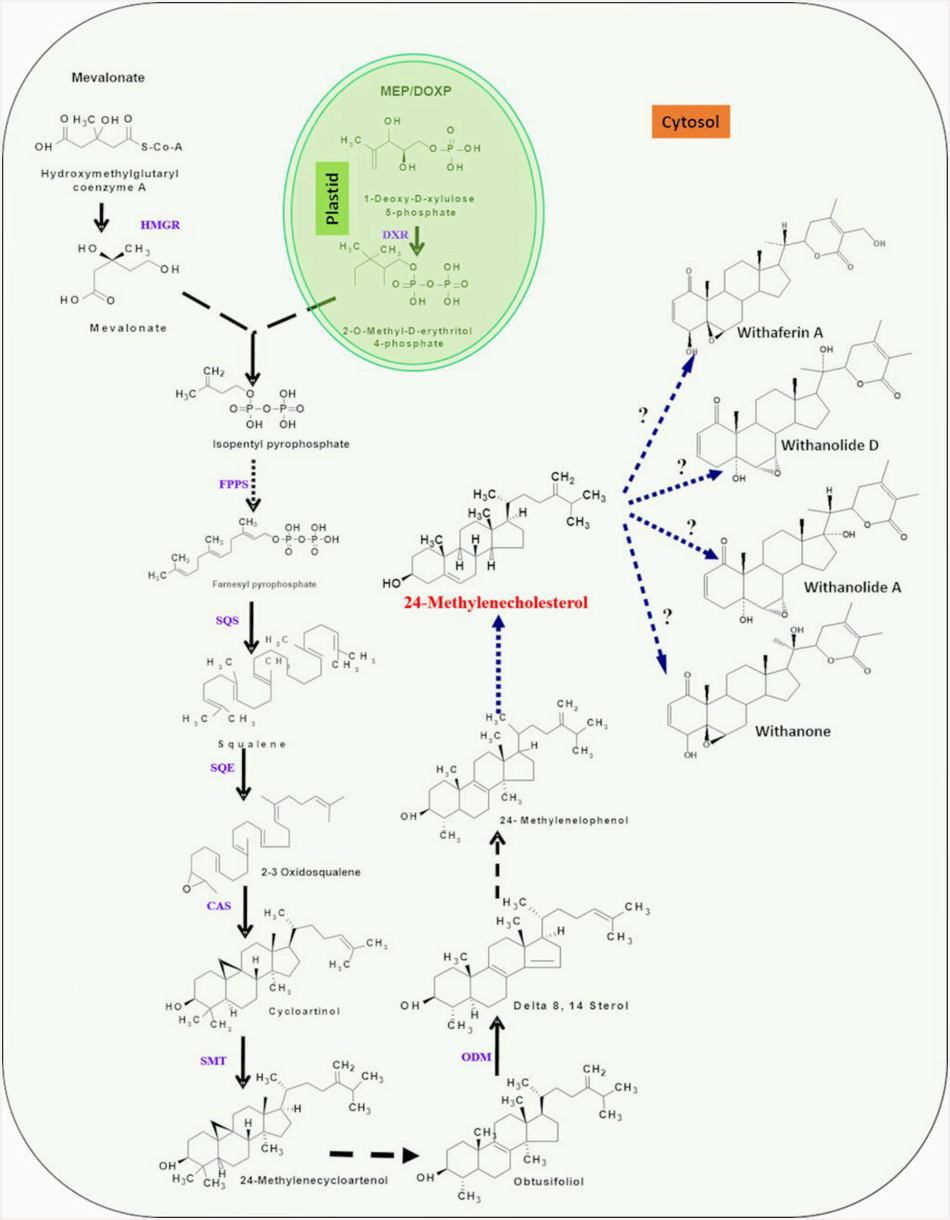
**An overview of putative withanolide biosynthesic pathway**. DXP, 1-deoxy-D-xylulose 5-phosphate; HMBDP, 1-hydroxy-2-methyl-2-(E)-butenyl 4-diphosphate; IPP, Isopentylpyrophosphate; DMPP, Dimethylalyl diphosphate; IPP isomerase, Isopentylpyrophosphate isomerase; FPPS, farnesyldiphosphate synthase; SQS, Squalene synthase; SQE/CPR, Squalene epoxidase/cytochrome P450 reductase; CAS, Cycloartenol synthase; SMT-1, Sterol methyl transferase/cytochrome P450 reductase; ODM/CPR, Obtusifoliol-14-demethylase/cytochrome P450 reductase. First three highlighted (yellow) steps indicating involvement of P450 monooxygenases and CPR. Single dark arrows represent one step, two or more dark arrows represent multiple steps and dashed arrow represents unknown steps.

Chemically, withanolides are 30-carbon compounds called triterpenoids. Triterpenoid backbone, like other terpenoid compounds is biosynthesized by metabolic pathway requiring isoprene units (isopentenylnpyrophosphate; IPP and dimethyl allyl pyrophosphate; DMAPP) as precursors. Therefore, isoprenogenesis could be one of the key upstream metabolic processes governing flux of isoprene units for synthesis of metabolic intermediate(s) of triterpenoid pathway committed to withanolide biosynthesis (Bhat et al., 2012). Dual autonomous pathways for the isoprenoid precursor biosynthesis co-exist in plant cell including the classical cytosolic mevalonic acid (MVA) pathway and the alternative route, plastidial methylerythritol phosphate (MEP) pathway (Newman and Chappell, 1999). Plastidial MEP pathway synthesizes IPP and DMAPP required for production of photosynthesis associated isoprenoids (carotenoids and side chains of chlorophylls, plastoquinones, and phylloquinones) and hormones (gibberellins and abscisic acid). In plants, MVA-derived isoprenoid end products comprise of sterols (modulators of membrane architecture and plant growth and developmental processes), brassinosteroids (steroid hormones), dolichol (involved in protein glycosylation), and the prenyl groups necessary for protein prenylation and cytokinin biosynthesis (Lichtenthaler, 1999; Rodriguez-Concepción et al., 2004). However, now there is growing evidence that a considerable cross-talk between the two pathways of isoprenogenesis exists and exchange of isoprene units may occur at different sub-cellular locations (Chaurasiya et al., 2012). A brief overview of the two metabolic routes for isoprene biosynthesis is elucidated under following sub-sections (Figure 2).

### Mevalonate pathway

MVA pathway involves seven enzymes for the synthesis of precursor molecules i.e., IPP and DMAPP for terpenoid biosynthesis. First step involves the condensation of two molecules of acetyl-CoA into acetoacetyl (AcAc)-CoA by the enzyme AcAc-CoA thiolase (Vranová et al., 2013) to form 3-hydroxy-3-methylglutaryl-coenzyme (HMG-CoA). In the second step, HMG-CoA synthase facilitate condensation of AcAc-CoA with one molecule of acetyl-CoA to form HMG-CoA (Nagegowda et al., 2004). Subsequently, HMG-CoA reductase (HMGR), a nicotinamide adenine dinucleotide (phosphate)-dependent (NAD(P)H) enzyme that catalyzes a double reduction reaction involving four electron transfers, results in biosynthesis of mevalonate from HMG-CoA (Benveniste, 2002). Conversion of mevalonate to IPP encompass two phosphorylations and decarboxylation events involving mevalonate kinase, phosphomevalonate kinase, and mevalonate diphosphate decarboxylase enzymes, respectively. Further, IPP derived from cytosolic MVA pathway, is acted upon by isopentenyl diphosphate isomerase, a divalent, metal ion-requiring enzyme, to form DMAPP (Hunter, 2007).

### Methylerythritol phosphate pathway

First step of the MEP pathway is catalyzed by 1-deoxy-D-xylulose 5-phosphate synthase (DXS), converting the precursors pyruvate and glyceraldehyde 3-phosphate into 1-deoxy-Dxylulose 5-phosphate (DXP; Sprenger et al., 1997; Cordoba et al., 2009). DXP reductoisomerase transforms DXP into MEP which is further converted to 1-hydroxy-2-methyl-2-(E)-butenyl 4-diphosphate by the consecutive enzymatic action of 2-C-methyl-D-erythritol 4-phosphate cytidylyltransferase, 4-diphosphocytidyl-2-C-methyl-D-erythritol kinase, 2-C-methyl-D-erythritol 2,4-cyclodiphosphate synthase, and (E)-4-hydroxy-3-methylbut-2-enyl diphosphate synthase (HMBPP). The last step is the branching of HMBPP to IPP and DMAPP catalyzed by the simultaneous enzymatic action of a single enzyme, (E)-4-hydroxy-3-methyl but-2-enyl diphosphate reductase (HDR). Although, HDR in MEP pathway produces both IPP and DMAPP, albeit at 85:15 ratio, the plastid localized isopentenyl diphosphate isomerase is involved in substrate optimization by catalyzing IPP isomerization. The head-to-tail condensation of IPP leads to formation of farnesyl pyrophosphate (FPP). FPP is the main precursor for triterpenoids (Kuzuyama, 2002; Chaurasiya et al., 2012) and is synthesized by catalytic action of the enzyme farnesyl diphosphate synthase (FPPS). It serves as a substrate for first committed reaction of several branched pathways leading to synthesis of compounds that are essential for plant growth and development as well as of pharmaceutical interest (Newman and Chappell, 1999). FPPS catalyzed reaction occurs in two consecutive steps; condensation of IPP with DMAPP to form 10-C intermediate geranyl diphosphate (GPP) and condensation of GPP with another molecule of IPP which results into 15-C FPP (Ohnuma et al., 1996). Squalene is believed to be a metabolic intermediate for biosynthesis of diverse triterpenoids. Its biosynthesis takes place by a reaction requiring squalene synthase enzyme that catalyzes head-to-head condensation of two molecules of FPP in NADPH dependent manner to produce squalene. The squalene undergoes epoxidation at one of its terminal double bonds by squalene epoxidase yielding squalene 2,3-epoxide. A ring closure reaction acting upon squalene 2,3-epoxide, catalyzed by cycloartenol synthase enzyme leads to the biosynthesis of cycloartenol that may further get converted into a variety of different steroidal triterpenoidal skeletons (Zhao et al., 2013; Dhar et al., 2014). The 24-methylenecholestrol, believed to be biosynthesized from cycloartenol has been proposed to be a central intermediate in the metabolic route toward withanolide biosynthesis. The hydroxylation at C-22 and δ-lactonization between C-22 and C-26 of 24-methylenecholestrol are believed to be important reactions leading to withanolides biosynthesis (Figure 2). In addition, it has also been suggested that α, β-unsaturated ketone in ring A of common withanolides may be produced through the sequential reactions. There is a scanty understanding with respect to enzymes and genes involved in these downstream reactions and withanolide biosynthesis.

## *De novo* tissue-specific withanolide biosynthesis

Phytochemical data generated over the years support considerable qualitative overlap of leaf and root withanolides in *W. somnifera*. Gradient of withanolide concentration with higher in leaves and lower in roots and radiotracer studies using 24-methylene cholesterol as a precursor also hints toward a possible import of withanolides from leaves to roots. Nevertheless, investigations reveal incorporation of 14C from [2–14C]-acetate and [U-14C]-glucose into WS-1 in *in vitro* cultured roots and native/orphan roots of *W. somnifera*. This study showed these primary metabolites being integrated into WS-1, thus indicative of *de novo* synthesis of root-specific WS-1 from primary isoprenogenic precursors rather than hinting toward an import from leaves (Sangwan et al., 2007).

Another interesting study reveals the analogy in the qualitative and quantitative profile of withanolide accumulation in leaf and root tissues of two morpho-chemovariants, suggesting *de novo* tissue-specific withanolide biosynthesis. Two genetic stocks designated as WS-Y-08 (25-30 cm tall with yellow berries) and WS-R-06 (100–125 cm tall with red berries) showed appreciable variation in their competence to synthesize and accumulate different withanolides. Three major withanolides viz. WS-1, withanone (WS-2), and WS-3 were assayed from leaf and root tissues harvested at five developmental stages. Additionally, transcript profiles of five withanolide biosynthetic pathway genes namely squalene synthase (*WsSQS*; GenBank Accession Number GU474427), squalene epoxidase (*WsSQE*; GenBank Accession Number GU574803), cycloartenol synthase (*WsOSC/CS*; GenBank Accession Number HM037907), cytochrome P450 reductase 1 (*WsCPR1*; GenBank Accession Number HM036710), cytochrome P450 reductase 2 (*WsCPR2*; GenBank Accession Number GU808569; Table 1) were also examined in the harvested tissues. The aim was to compare gene expression with that of metabolite flux at different phenophases. Relative transcript abundance demonstrated significant deviation in leaf and root tissues that was mostly parallel with the divergence in withanolide pool. Leaves in comparison to roots showed elevated gene expression in corroboration with improved concentration of all the three withanolides. This relative dynamics of all the three withanolides at quantitative and qualitative levels in the two withanolide richest tissues possibly indicate toward *de novo* tissue-specific biosynthesis (Dhar et al., 2013).

**Table 1:** **Putative pathway genes from *Withania somnifera***.

**Genes involved**	**Expression system used**	**Abiotic/Biotic factors used**	**Effect on gene/Expression and withanolide levels**	**Reference**
3-Hydroxy-3-methylglutaryl coenzyme A reductase	*E. coli*	Salicylic acid, methyl-jasmonate, and mechanical injury		http://link.springer.com/article/10.1007%2Fs00709-012-0450-2
*WsSGTL1*/Sterolglycosyltrnasferases	*A. thaliana*	Salt, heat, and cold	Increase in *Ws*SGTL1 HSP70, HSP90, RD29, SOS3, and LEA4-5 levels	http://www.ncbi.nlm.nih.gov/pubmed/23646175
1-Deoxy-D-xylulose-5-phosphate synthase		Salicylic acid, methyl jasmonate, mechanical injury	Maximum expression in flower and young leaves	http://www.ncbi.nlm.nih.gov/pubmed/22526204
1-Deoxy-D-xylulose-5-phosphate reductase		Salicylic acid, methyl jasmonate, mechanical injury	Differentially expressed in different tissues	http://www.ncbi.nlm.nih.gov/pubmed/22526204
*WsCYP98A*	*E. coli*	Methyl jasmonate salicylic acid, gibberellic acid	Methyl jasmonate salicylic acid increased and gibberellic acid decreased *Ws*CYP98 expression	http://www.biomedcentral.com/1472-6750/14/89
			WS-3 accumulation increased upon methyl jasmonate elicitation; gibberellic acid treatment decreased WS-3 and WS-1 accumulation	
*WsCYP76A*	*E. coli*	Methyl jasmonate, salicylic acid, gibberellic acid	Methyl jasmonate, salicylic acid as positive regulators and gibberellic acid acted as a negative regulator for *Ws*CYP98A	http://www.biomedcentral.com/1472-6750/14/89
			Methyl jasmonate elicitation increased WS-3 accumulation; gibberellic acid decreased WS-3 and WS-1 accumulation	
*WsCPR1*	*E. coli*	Methyl jasmonate, salicylic acid	The expression remained unchanged in *Ws*CPR1; increase in WS-1 levels, less in WS-2 levels	http://www.ncbi.nlm.nih.gov/pmc/articles/PMC3578826/
*WsCPR2*	*E. coli*	Methyl jasmonate, salicylic acid	The treatments with methyl jasmonate, salicylic acid resulted in induction of *Ws*CPR2 the expression levels	http://www.ncbi.nlm.nih.gov/pmc/articles/PMC3578826/
*WsSQS*	*E. coli*	Methyl jasmonate, salicylic acid	Withanolides were regulated by methyl-jasmonate, salicylic acid and 2,4-D, treatments	http://www.ncbi.nlm.nih.gov/pubmed/22425978
*WsSQE*	*E. coli*	Methyl jasmonate, salicylic acid	Biosynthesis of withanolide was up-regulated by methyl-jasmonate, salicylic acid and 2,4-D	http://www.ncbi.nlm.nih.gov/pubmed/23065254
Oxidosqualene cyclases (OSCs) *WsOSC/LS, WsOSC/BS, WsOSC/CS*	*S. pombe*	Methyl jasmonate, gibberellic acid and yeast extract	Upon methyl jasmonate treatment transcript levels of *Ws*OSC/LS were down regulated, unchanged in case of *Ws*OSC/CS, and upregulated in case of Bs. YE down regulated *Ws*OSC/LS and *Ws*OSC/BS transcripts. gibberellic acid induced *Ws*OSC/BS and down regulated *Ws*OSC/LS transcripts	http://www.jbc.org/content/early/2014/04/25/jbc.M114.571919
*WsDWF1*	*E. coli*	Methyl jasmonate, gibberellic acid and 2,4-D	The treatments with methyl jasmonate, 2,4-D and salicylic acid resulted increased expression of *Ws*DWF1	
			Transcripts	
*WsFPPS*		Methyl jasmonate, salicylic acid, mechanical injury	Maximum expression was found in flower and young leaf and NMITLI-101 chemotype. Significant elevation in response to salicylic acid, methyl jasmonate and mechanical injury	http://link.springer.com/article/10.1007%2Fs10725-011-9578-x#page-1

## Gene elucidation of withanolide biosynthetic pathway

Synthesis of withanolides chemically, is complex due to the stereo-chemical ring closure, occurrence of chiral centers, rigid trans-lactone groups, and high energy epoxy ring (Neumann et al., 2008). Thus, making synthetic production economically unworkable due to minimal yields at high costs. Therefore, it demands viable alternatives for the production of withanolides in large quantities for commercial exploitation. Genetic manipulation with genes encoding enzymes involved in withanolide biosynthesis seems to be a viable approach that may be useful in developing genotypes of *W. somnifera* with enhanced levels of withanolides as well as for withanolide producing alternative microbial/yeast hosts. This whole approach entails expression of metabolic circuitries in heterologous host(s). It requires comprehensive knowledge about complete genetic architecture of withanolide biosynthesis including enzymatic and regulatory genes of the pathway. Nevertheless, withanolide biosynthetic pathway still remains in its putative stage at molecular level. Hence, it is of fundamental research value to elucidate the withanolide biosynthetic pathway encompassing the delineation of regulatory aspects of their biosynthesis also. Inception of pathway exploration has gained much interest by researchers over the past few years vis-à-vis withanolides. There have been several endeavors by many workers including comprehensive investigations carried out by our group related to isolation, cloning, characterization and regulation of several pathway genes, promoters, elicitations in corroboration with metabolite production and substrate pool diversion for enhanced withanolide yields. These different aspects are covered in the ensuing text.

## 3-hydroxy-3-methylglutaryl coenzyme A reductase

3-Hydroxy-3-methylglutaryl coenzyme A reductase (3-HMGR) is a NADH dependent rate limiting enzyme involved in the conversion of 3-hydroxy-3-methylglutaryl coenzyme A (HMG-CoA) into mevalonic acid; chief precursor of IPP and DMAPP in the isoprenoid biosynthetic pathway. Plant HMGR have been located in the subcellular organelles like endoplasmic reticulum (ER), mitochondria, and plastids with catalytic domains present in cytosolic portions of cell (Kim et al., 2015). Owing to its rate limiting nature this enzyme is a target of various cholesterol lowering drugs such as statins (Istvan and Deisenhofer, 2001). Functionally it has been characterized in both mammalian and plant systems. In plants HMGR is encoded by multigene family which exhibits variegated temporal and spatial expression pattern. In *Arabidopsis* HMGR isozymes are encoded by *HMGR-1* and *HMGR-2*. Loss of function of *HMGR-1* leads to the generation of dwarf phenotype, sterility and senescence, corresponding to the diminished sterol biosynthesis (Kim et al., 2015). Considering its important role in isoprenoid biosynthesis HMGR has been explicitly studied and characterized in many plant and other plant species which include *Catharanthus roseus, Ginkgo biloba, Taxus media, Salvia miltiorrhiza*, etc. (Akhtar et al., 2013).

Studies carried out have shown the existence of a relationship between withanolide biosynthesis and HMGR-1 expression. In this study a positive correlation between high transcript levels of HMGR and optimum accumulation of withanolides was found in the root tissue of *W. somnifera* (Table 1; Akhtar et al., 2013). It may be attributed to the enhanced biosynthesis of substrate pool or precursors like IPP and DMAPP for various biosynthetic pathways including withanolide biosynthetic pathway. Also reports of *HMGR-1* mutants generating diminished sterol content in *A. thaliana* and mevinolin directed inhibition of HMGR leading to significant decrease in total ginsenoside in *P. ginseng* adventitious roots (Kim et al., 2015) suggest a link between *HMGR-1* expression and sterol biosynthesis. Positive elicitation i.e. increased expression of *WsHMGR* in response to salicylic acid (SA) and methyl jasmonate (MJ) indicates presence of *cis* regulatory elements in promoter region which may regulate the expression of *WsHMGR* in various biosynthetic pathways including withanolide biosynthetic pathway. Young leaves expressed high levels of *WsHMGR* transcripts than in mature leaves (Chaurasiya et al., 2007). These results correlated positively with the enhanced levels of withanolide production in young leaves relative to that of mature leaves of *W. somnifera*. HMGR has been demonstrated as an accelerator of isoprenoid biosynthesis. There was two-fold increase in the biosynthesis of β-carotene in *E. coli* (Akhtar et al., 2013) by tandem expression of *WsHMGR* and PAC Beta gene. It suggested that HMGR provides enhanced progenitor substrate pool for various biosynthetic pathways including withanolide biosynthesis.

## 1-deoxy-D-xylulose-5-phosphate reductoisomerase and 1-deoxy-D-xylulose-5-phosphate synthase

Plausibly, withanolides, the signature secondary metabolites of *W. somnifera*, are biosynthesized through metabolic deviation from sterol pathway at the level of 24-methylene cholesterol (Sangwan et al., 2004). Isoprenoid precursor for the same is synthesized by MVA pathway and MEP pathway wherein MEP pathway is the plastid-derived alternative route for isoprenoid biosynthesis. MEP pathway contributes about 30% in biosynthesis of withanolide precursor isoprenoids (Tuli et al., 2009) in which the first step is a condensation of pyruvate with Dglyceraldehyde-3-phosphate to form 1-deoxy-D-xylulose-5-phosphate (DXP), catalyzed by DXP synthase (DXS). DXP acts as a precursor for IPP and DMAPP biosynthesis (Julliard and Douce, 1991; Julliard, 1992; Himmeldirk et al., 1996). Subsequently, conversion of DXP to MEP, is catalyzed by DXP reductoisomerase (DXR). Though, DXR is the opening committed step for terpenoid biosynthesis through the MEP, DXS, the first enzyme of this pathway, also is significant for isoprenoid biosynthesis in several organisms, including bacteria and plants (Estévez et al., 2001; Guevara-García et al., 2005). To understand the significance of MEP pathway in isoprenoid biosynthesis in *Withania*, full-length cDNAs of *WsDXS* and *WsDXR* were cloned and characterized (Table 1). Saptial expression analysis revealed elevated level of *WsDXS* and *WsDXR* transcripts in young leaf that correlates with the reported enhanced rates of withanolide biosynthesis in young than in mature leaf of *W. somnifera* (Chaurasiya et al., 2007). Lower root expression of *WsDXS* and *WsDXR* than leaf also hinted toward their plastid-localization. This hints toward root being less active site for isoprenoid biosynthesis utilizing substrate from MEP pathway. Though, leaves and roots both are involved in withanogenesis independently, leaves are possibly the prime site for the same. Further, gene expression level of *WsDXR* and *WsDXS* were corroborated with qualitative and quantitative withanolide variations in chemotypes NMITLI-101, NMITLI-118, and NMITLI-135 possessing WS-3, WS-2, and withanolide D as the main withanolides in leaf tissue and withanolide A as the main withanolide in the root tissue. *WsDXS* expressed maximally in leaf of NMITLI-118 and 135 which was concurrent with high accretion of withanolides in these chemotypes. Conversely, expression of *WsDXR* transcript was observed approximately equivalent in leaves of all three chemotypes. Consequently, indicating that enzymes contribute in similar manner during isoprenoid biosynthesis in different chemotypes (Gupta et al., 2013a).

## Farnesyl diphosphate

MVA and MEP pathway are attributed with most of the bioactive molecules synthesized in *Withania*. In these biosynthetic routes, farnesyl diphosphate (FPP), acts as a substrate for foremost committed reaction of numerous branched pathways and is synthesized by the enzyme farnesyl diphosphate synthase (FPPS) in two successive steps. Firstly, condensation of IPP with DMAPP structures 10-C intermediate geranyl diphosphate (GPP). Further condensation of GPP with another molecule of IPP forms FPP. FPPS is an important enzyme for biosynthesis of isoprenoid that synthesize sesquiterpene precursors for vital metabolites including sterols, dolichols, ubiquinones, and carotenoids in addition to substrates for farnesylation and geranylgeranylation of proteins. Overexpression of ginseng farnesyl diphosphate synthase in *Centella asiatica* hairy roots also enhanced phytosterol and triterpene biosynthesis (Kim et al., 2010). FPPS as well caters an important role in incipient steps of triterpenoid precursor production related to withanolide biosynthesis. Consequently, highlighting the significance of FPPS in any pathway engineering attempt for enhancing a desired isoprenoid of primary or secondary importance. FPPS has been characterized from a range of different plant species like *Arabidopsis* (Closa et al., 2010), *Artemisia* (Matsushita et al., 1996), *Hevea* (Takaya et al., 2003), maize (Cervantes-Cervantes et al., 2006), etc. As a step toward elucidating the significance of FPPS as the key entry point enzyme of the withanolide biosynthesis in *W. somnifera*, full-length FPPS cDNA was isolated and characterized as it constitutes a key step en route to biosynthesis of the progenitor(s) of withanolide biosynthesis (Table 1). Significance of *WsFPPS* gene in synthesis of sesqui- and higher isoprenoids counting the metabolites obtained from them was displayed by the constitutive expression of *WsFPPS* in all parts of the plant. Higher expression level of *WsFPPS* in young leaf as compared to the mature leaves corroborated with the reported enhanced withanolide biosynthesis in young leaf of *W. somnifera* (Chaurasiya et al., 2007; Gupta et al., 2011).

## Squalene synthase

Squalene synthase (SQS) (farnesyl diphosphate: farnesyl diphosphate farnesyl transferase, EC 2.5.1.21) catalyzes one of the initial enzymatic steps of phytosterol biosynthetic pathway, facilitating condensation of two farnesyl pyrophosphate molecules to squalene. SQS routes carbon flux from isoprenoid pathway toward the phytosterol biosynthesis resulting in formation of endproducts like brassinosteroids, withanolides, and triterpenoids (Abe et al., 1993). SQS has been reported to be active in ER, it anchors to it via carboxyterminal portion. The cytosolic portion is anchored via amino terminal of protein (Robinson et al., 1993). SQS plays a key regulatory function in phytosterol biosynthesis. Overexpression of SQS genes in *P. ginseng* (Lee et al., 2004) and *Eleutherococcus senticosus* (Seo et al., 2005) led to the improved accretion of phytosterols and triterpenes thus highlighting the significant regulatory function of SQS in plants. Although ample evidence is available regarding the role of SQS in phytosterol biosynthesis, scanty is identified about the biosynthetic pathway of withanolides, genes involved in the withanolide biosynthesis and regulatory elements of promoter region governing the gene expression in *W. somnifera*. Thus, for pathway intensification leading to enhancement of withanolide accumulation in *W. somnifera* Bhat et al. (2012) investigated the significance of squalene synthase in withanolide biosynthesis. Characterization of *WsSQS* including tissue specific expression analysis and regulatory studies at promoter level substantiated *WsSQS* as an imperative gene target involved in withanolide biosynthesis (Table 1). *WsSQS* demonstrated increased expression pattern in leaves that was in consonance with the elevated production of withanolides in leaves of *W. somnifera*. Additionally, biosynthesis of withanolides and mRNA abundance of *WsSQS* were enhanced through diverse signaling molecules including methyl-jasmonate, salicylic acid, and 2,4-D that was regular with the expected results of *WsSQS* promoter. Thus, hinting toward the unraveling of a key committed step of the withanolide biosynthetic pathway (Bhat et al., 2012).

## Squalene epoxidase

Squalene epoxidase (SE) (EC 1.14.99.7) is a rate limiting enzyme in the sterol biosynthetic pathway, catalyzing the conversion of squalene into 2,3-oxidosqualene by carrying out stereospecific epoxidation reaction (Ryder, 1992; He et al., 2008). This enzyme requires cytosolic (S105) fraction, molecular oxygen, NADPH-cytochrome c reductase, NADPH and flavine adenine dinucleotide (FAD) for its activity (Abe et al., 2007). SQE, a lightly bound FAD flavin, attains electrons from NADPH-cytochrome reductase, instead of binding the nicotinamide cofactor directly which differentiates it from other flavin mono-oxygenases. It is mainly located in the endoplasmic reticulum and lipid droplets but protein located in the endoplasmic reticulum is active (Leber et al., 1998). Additionally, SQE activity can result in the formation of 6,7-oxidosqualene, 10,11-oxidosqualene, and dioxidosqualene (Bai and Prestwich, 1992). SQE has been found to be main precursor for all identified angiosperm cyclic triterpenoids, that comprise membrane sterols, non-steroidal triterpenoids, brassinosteroid, and phytohormones. Being a rate limiting enzyme SQE has a cascading influence on the upregulation of downstream genes (Han et al., 2010). Thus, genetic manipulation of SQE in host plant offers an exciting prospect for production of desired therapeutic triterpenoid molecules (Takemura et al., 2010). This has been demonstrated in *P. ginseng* for heightened synthesis of triterpene saponins and phytosterols using squalene synthase (Lee et al., 2004). Against this backdrop, Razdan et al. (2013) has reported the substantial notice to comprehend the regulatory function of SE in withanolides biosynthesis. Toward this goal, *WsSQE* gene along with its promoter was isolated from *W. somnifera* and several cis-regulatory elements of promoter region were revealed (Table 1). This paves a way to recognize the regulatory function of SQE in withanolides biosynthesis as *WsSQE* also displayed maximum expression in withanolide richest leaf tissue. Keeping in view the prospect of pathway intensification, significance of *WsSQE* as a robust target lies in its rate limiting nature (Razdan et al., 2013). This significance can be utilized with an efficient *Agrobacterium* mediated transformation system in *W. somnifera* for homologous modulation of withanolide biosynthesis.

## Cycloartenol synthase

Withanolides are synthesized *via* both MVA and MEP pathways which direct the flux of the isoprene (C5) units for the synthesis of triterpenoid pathway intermediates which are further committed to withanolide biosynthesis. Sterols, withanolides, and various triterpenoids are synthesized through a common 30-carbon intermediate 2,3-oxidosqualene in a highly regio and stereo-specific step catalyzed by a family of genes called oxidosqualene cyclases (OSCs) (Phillips et al., 2006). Plants produce a variety of triterpenoid skeletons structured by numerous OSC enzymes broadly belonging to two groups i.e., protosteryl and dammarenyl cations based on the nature of their supposed catalytic intermediates (Phillips et al., 2006). Both these cations as backbones impart discrete stereochemistry and ring configurations to various triterpenes. The protosteryl cation with chair-boat-chair (C-B-C) configuration forms cycloartenol, lanosterol, cucurbitadienol, and parkeol tetracyclic triterpene structures. The majority of the pentacyclic triterpenes are however, derived from the dammarenyl cation by D-ring expansion to form lupeol or further E-ring expansion to form β-amyrin (Xu et al., 2004). Similarly, partitioning of the common substrate, 2,3-oxidosqualene in *W. somnifera* takes place between OCS-cycloartenol synthase [(S)-2,3-epoxysqualene mutase (cyclizing, cycloartenol forming), EC 5.4.99.8] (CAS) and other OSCs. CAS forms cycloartenol, a pentacyclic triterpene with nine chiral centers and functions as the precursor to phytosterols and apparently to withanolides and other diverse OSCs structure diverse triterpenoids like lupeol, beta amyrin, etc., (Rees et al., 1969). This partitioning constitutes a metabolic branching point leading to the division of 2,3-oxidosqualene between sterol/withanolides and range of triterpenoids (Figure 1). Thus, making genes covering the branches of these sub-dividing point prospective candidates for perturbation. Such manipulations hold significant possibility of impacting respective branch flux by redirecting the precursor reservior in the direction of preferred secondary compound and concurrently reduce the flux via competitive biosynthetic routes. Against this backdrop, in *W. somnifera* three members of OSC superfamily viz. β-amyrin synthase (*WsOSC/BS*; GenBank Accession Number JQ728553), lupeol synthase (*WsOSC/LS*; GenBank Accession Number JQ728552), and *WsOSC/CS* covering three branches of a sub-dividing junction leading to withanolides, sterols and a suite of triterpenoids have been characterized (Table 1). Regulatory studies of WsOSCs involving plant-derived methyl jasmonate and giberrelic acid and microbe-derived yeast extract elicitations displayed differential transcriptional and translational profiles that were clearly reflected in visible variations in withanolide quantity. MJ elicitation considerably augmented WS-3 accretion over a period of 48 h that was in consonance with studies involving MJ-induced up-regulation of *WsSQS, WsSQE*, and *WsCPR2* mRNA also led to enhanced withanolide accumulation. It may be attributed to increased synthesis of 2,3-oxidosqualene produced by induced upstream genes. As a consequence, *WsOSC/CS* is able to utilize an increased precursor pool for withanolide biosynthesis. Although the OSC mRNA expression model in case of gibberellic acid (GA_3_) coincided with MJ treatment, the total withanolide accumulation demonstrated a regular drop with increasing time course. This may be attributed mainly to the decrease in *WsOSC/CS* protein concentration as evident from the Western blot study. Nevertheless, transcript abundance of *WsOSC/BS* showed a rise that hinted toward the decrease in the total substrate availability for *WsOSC/CS*, but at the protein level, *WsOSC/BS* expression declined with increasing time intervals, thus possibly substantiating the drop in WS-3 concentration caused by decreased *Ws*OSC/CS protein availability. Interestingly, microbe-derived exogenous yeast extract (YE) elicitor played a role of negative regulator for the two competitive OSCs of *WsOSC/CS* (*WsOSC/BS* and *WsOSC/LS*) at both the protein and mRNA levels, whereas *WsOSC/CS* showed no change in its transcript or protein expression in response to YE. However, there was significant increase in withanolide concentration with YE in comparison with MJ treatment. The down-regulation of *WsOSC/BS* and *WsOSC/LS* is possibly indicative of differential channeling of common substrate among the three branch OSCs. Plausibly, this leads to rearrangement of metabolic fluxes wherein bulk of 2,3-oxidosqualene substrate pool shifts toward *WsOSC/CS*, leading to much improved withanolide yields. The characterization and validation of *WsOSCs* seem important for strategizing the enhanced production of withanolides (Dhar et al., 2014).

## Cytochrome P450 reductase and monooxygenases

Cytochrome P450 enzymes, member of one of the key functionally diverse protein super-families. It is essential in a variety of metabolic molecular circuitries. P450s are heme thiolate-proteins, catalyse enormously varied reactions like hydroxylations, dealkylations, sulfoxidations, epoxidations, reductive dehalogenations, peroxidations, and different types of isomerization for the synthesis of a number of primary and secondary metabolites indispensable for plant growth and development (Guengerich, 2001a; Hrycay and Bandiera, 2012). P450 monooxygenases comprise a substrate explicit class of enzymes which are highly regio and stereo-specific. Gene annotation has shown that about 1% of the entire genes in the plant's genome are cytochrome P450s. *Arabidopsis* genome contains 244 genes and 28 pseudo-gene representing cytochrome P450s (Nelson et al., 2004). P450s are ER localized with their catalytic functioning depending on source of electrons through NADPH cytochrome P450 reductase (CPR: diflavoenzyme).

CPRs (EC 1.6.2.4) possess a N-terminal positioned flavin mononucleotide (FMN) binding domain linked to NADPH binding domain *via* flavin adenine dinucleotide (FAD) domain are membrane bound proteins localized to ER. These are responsible for shuttling electrons obtained from NADPH through FAD and FMN domains into the heme iron-center of the various P450 enzymes. CPR genes have been isolated from numerous species of yeast, animals and insects. Between all, only one form is identified to network with several P450s (Simmons et al., 1985). Conversely, many CPR paralogs varies based on the vascular plant species. Ro et al. (2002) have categorized CPRs into Class I and class II groups on the basis of N-terminal anchoring sequences. *CPR1* belonging to class I expresses constitutively whereas *CPR2* in class II, is expressed in stress or on wound elicitation. Plants encode several CPRs that reflects the range of P450s (Werck-Reichhart et al., 2000; Feldmann, 2001) and their role in primarily confronting the elevated requirement of electrons during various stresses or varied expression at different plant developmental stages (Mizutani and Ohta, 2010).

Additionally, P450 monooxygenases also correspond to a highly regio and stereo-specific class of fixed substrate-specific enzymes that play a decisive function in secondary metabolism and mostly aid in functionalizing core structures of molecules like withanolides. Due to their regio and stereo-specific catalyzing flexibility, these are possible targets for industrial biocatalysis. P450s have been useful in industry for the examination of new medicine, drugs or xenobiotics (Guengerich, 2002, 2011; Miners, 2002). P450s are considered as the most versatile biological catalysts in nature due to the notable diversity of chemical reactions catalyzed and vast substrates attacked (Sono et al., 1996; Guengerich, 2001b; Coon, 2005). Consequently, accentuating their identification and characterization for biosynthetic pathway elucidation. Due to the polyphyletic nature of plant P450s these are commonly categorized in two main clades, A-type and non-A-type. Plant specific metabolism and biosynthesis of diverse natural products involves A-type P450s (Bak et al., 2011). The molecular and biochemical characteristics of cytochrome P450 reductases and monooxygenases in relation to pathway engineering, emphasizes the significance of this family of genes as robust gene targets of both primary and secondary metabolite biosynthesis.

Molecular and biochemical studies started to reveal biosynthetic routes for several withanolides in *W. somnifera* encompasses this important family of genes. Two A-types P450 *WsCYP98A* (GenBank Accession Number HM585369) and *WsCYP76A* (GenBank Accession Number KC008573) and two paralogs of cytochrome P450 reductases from *W. somnifera* have been isolated, sequenced and heterologously expressed in *E. coli* (Rana et al., 2013, 2014) (Table 1). All the four CYPs at transcript level are spatially regulated displaying variance in tissue specificity. Expression of *WsCPR2* is coincident with the elevated withanolides content in *W. somnifera* leaves. This probably indicates involvement of *WsCPR2* to confront increased reductive demand of diverse P450 monooxygenases for carrying the withanolides biosynthesis. Elicitation studies showed exogenous elicitors acting as both positive and negative regulators of mRNA transcripts of *Ws* monooxygenases and reductases. MJ and SA resulted in abundant *WsCYP98A* and *WsCYP76A* expression. Increased mRNA levels also agreed with the high accumulation of elicitation driven withanolides biosynthesis. There was appreciable enhancement in WS-1 and WS-3 in response to elicitations. In MJ treated samples, there was a 2.5-fold increase in WS-1 and a significant 4.2-fold enhancement of WS-3. SA treated samples showed marked increase of 1.7- and 3.2-fold in WS-1 and WS-3, respectively. Conversely, GA_3_ decreased the expression of *WsCYP98A* as *WsCYP76A* in addition to the gradual decline in WS-3 whereas WS-1 showed an increase up to 24 h followed by a decrease in WS-1 after 48 h (Rana et al., 2014). Whereas, MJ and SA elicitation induced only *WsCPR2* reductase while as *WsCPR1* expression showed no change along with significant increase in WS-1 and WS-3 (Rana et al., 2013).

Additionally, four more CYP genes from *W. somnifera* christened as *WS*CYP93Id, *WS*CYP93Sm, *WS*CYP734B, and *WS*CYP734 belonging to CYP83B1 and CYP734A1 family and CYP71 and CYP72 clans, also displayed variance in expression in different tissues and in response to different treatments. Interestingly, all the four CYPs showed maximal expression in leaf tissue that was co-incident with the tissue specific secondary metabolite profile of *W. somnifera*. Furthermore, similar expression profile of *WS*CYP93Id, *WS*CYP93Sm, *WS*CYP734B and *WS*CYP734 in different *W. somnifera* chemotypes hinted toward their specialized role in biosynthesis of chemotype-specific metabolites. Light and auxin led to enhancement in expression of all the four CYPs. However, *WS*CYP734B displayed predominant responsiveness to light and auxin proposing its association with withasteroid/brassinosteroid regulation in planta. MJ and SA elicitations showed an increasing m-RNA abundance trend of the CYPs with increasing concentration of the elicitor. Functional validation of *WS*CYP93Id in *E. coli* using withanolides as substrates revealed change of withaferinA to a hydroxylated product. Relationship chart drawn for *WS*CYP93Id, *WS*CYP93Sm, *WS*CYP734B, and *WS*CYP734 sequences proposed that various withanolides like withanolide A, withanolide D, withaferin A, and withanone are possibly the ensuing yields of metabolic changes involving downstream biosynthetic genes by means of *WS*CYP enzymes (Srivastava et al., 2015).

Likewise, these results cumulatively, give a better understanding of the regulatory role of CPRs for increased production of withanolides by means of *Agrobacterium* mediated transformation system. This can lead to homologous intensification of overall metabolite flux with higher transcript levels of key regulatory genes correspondingly up-regulating downstream genes.

## Glucosyltransferases

Plant metabolism involves glycosylation as a common modification reaction that is perpetually related with secondary metabolism. Enzymes leading to glucoside formation are called as uridine diphosphate glycosyltransferases (UGTs), members of family 1 of the glycosyltransferase superfamily, which contains over 80 families of enzymes (Campbell et al., 1997; Coutinho et al., 2003) and their functioning involves transferring a uridine diphosphate (UDP)-activated glucose to an equivalent acceptor molecule. UGTs use UDP-activated sugars as donors and allocate their sugar moiety to many acceptors. Plant family 1 UGTs catalyse the glycosylation of surplus bioactive natural compounds. This is frequently the concluding step for biosynthesis of various natural products (Jones and Vogt, 2001), to improve their stability and solubility, and to facilitate storage and build-up in plant cells. Over the years, many UGT gene sub-families have evolved for molecular glycosylation (Vogt and Jones, 2000; Jones and Vogt, 2001). UGTs functioning in secondary metabolism carry a conserved 44 amino acid residue motif (60–80% identity) called as the plant secondary product glucosyltransferase box (PSPG), validated to include the UDP–sugar binding moiety (Hughes and Hughes, 1994; Offen et al., 2006). Nevertheless, UGTs show comparatively meager levels of sequence identity, particularly in the regions associated with acceptor binding. This might be important for the recognition of many acceptors and synthesis of huge number of products.

Many of the pharmacological properties of *W. somnifera* are attributed to its distinctive steroidal compounds, called glycowithanolides (Matsuda et al., 2001; Singh et al., 2001; Misra et al., 2005; Lal et al., 2006). However, there exists scarcity in the information related to the metabolic step(s) leading to their glyco-transformations due to non-availability of the relevant enzymes and genes.

The first study on sterol glucosyltransferases (SGTs) from *W. somnifera* reported three different (SGTs) *SGTL1, SGTL2*, and *SGTL3* comprising conserved SGT family domains (Table 1). Among these, SGTL1 was cloned in full length (DQ356887) and was found to be ubiquitously expressing in different parts of the plant. Deduced amino acid sequence of *SGTL1* showed the presence of transmembrane domains and preference for membrane sterol glucosylation. Moreover, partially purified recombinant SGT displayed specificity to sterols with hydroxyl group at C-3 position. Functional recruitment of *SGTL1* under environmental challenge(s) has also been reported in response to stress (Sharma et al., 2007). Further characterization of new sterol glucosyltransferases in *W. somnifera* can contribute to the disclosure of functions of various glycosterol in plants.

## DIMINUTO/DWARF1 (DIM/DWF1)

*DWF1* is an important gene of sterol biosynthesis and regulates the metabolic flux by carrying out isomerization, reduction, and epoxidation, respectively, of their immediate metabolic precursors. *DWF1* coding for key enzyme is involved in isomerization and reduction of 24-methylene cholesterol to campesterol and isofucosterol to sitosterol (Klahre et al., 1998). GFP-DIM-DWF1 transient expression studies have shown that *DWF1* is an integral membrane protein and is located to ER and not present in nucleus. Sequence homology with flavine adenine dinucleotide-binding domain (conserved in oxidoreductases) indicates that *DWF1* has possibly a catalytic rather than a regulatory function. Mutant studies have established the functional role of DIM protein in plant phytosterol biosynthesis. Other homologs of DWF1 have been characterized from *Arabidopsis thaliana, Pisum sativum, Zea mays*, etc. and their function has been elucidated by mutant studies

In one of the reports *Arabidopsis DIMINUTO*/*DWARF1* gene was found to encode for protein involved in phytosterol biosynthesis. Analysis of *A. thaliana* DIM mutant has revealed the dwarf phenotype with decreased fertility can be restored by addition of exogenous brassinolide. 24-Methylene cholestrol was found to accumulate in dim mutants. All of these mutants were deficient in campesterol, indicating toward the mutation in *DIM/DWF1* severely affects the phytosterol biosynthesis in plants (Klahre et al., 1998).

Reports of some site directed mutants have revealed complete loss of function of *DWF1* in plants due to loss of calmodulin binding, similarly complementation studies revealed that fractional loss of calmodulin binding led to partial dwarf phenotype (Du et al., 2009).

*DWF1* gene homologs have been isolated from pea and maize, and their function have been elucidated by mutant studies The mutants have been shown to accumulate 24-methylene cholesterol and are severely dwarfed with reduced fertility. Biochemical and mutant studies have revealed the function of DIM homolog in pea known as *LKB*. The homolog for DIM in pea is *LKB*, it was observed that mutation in DIM gene directly correlated with the altered phenotype such as decreased enzyme activity, truncated internodal length, epinastic leaves, and thickened stem, and accumulation of 24-methylene cholesterol. Upon exogenous application of brassinolide the phenotype reverted to the normal type. Northern analysis showed the ubiquitous presence of *LKB* gene in the plant. Role of *DWF1* is well-established in the brassinolide biosynthetic pathway. Analysis of dim mutants has revealed that DIM mutants lead to impaired biosynthesis of campesterol and consequent expression of a dwarf phenotype. 24-Methylene cholestrol is a shared precursor of both withanolide and brassinolide biosynthetic pathway (Choe et al., 1999).

DWF1 is a multifunctional enzyme which may carry out isomerization and reduction of post 24-methylene cholestrol intermediates upto withanolide biosynthesis, similar to its role in brassinolide biosynthesis. Increase in the transcript levels of *DWFI* in response to MJ, 2,4-dichlorophenoxyacetic acid (2,4-D) and SA and corresponding increase in the withanolide content indicates toward the involvement of *DWF1* in withanolide biosynthesis along with other biosynthetic pathway genes. Increase in the withanolide content may be due to the increase in the 24-methylenecholestrol substrate pool which may be a consequence of increased expression of upstream genes of MVA pathway such as *WsSQS, WsSQE, WsOSC/CS, WsOSC/LS, WsOSC/BS, WsCPR1*, and *WsCPR2* upon elicitation with abiotic factors such as MJ, 2,4-D, and SA. The presence of *cis* regulatory inducible elements present in the promoter region of genes involved in the withanolide biosynthetic pathway may be the reason behind the effect of elicitors.

Highest amount of DWF1 transcript level were found in leaves when compared to root and stalk tissues that corroborates well with the high levels of withanolides accumulation in leaves as explained in earlier studies. Also it supports the *de novo* synthesis of withanolides i.e., synthesis of withanolides in various parts of plant *via* complete metabolic pathway rather than transfer from any other plant tissue. The molecular cloning and characterization of DIM (GenBank Accession Number KP318739) from *W. somnifera* and transcript profiling data entails one of the recent studies carried out by the authors.

## Tissue-specific transcriptome analysis

More recently, for identification of putative biosynthetic pathway genes, there has been a huge swing toward the “omics” approach, that takes advantage of sequencing technologies to obtain genomic and transcriptomic sequence resource. For species where genomic data are unavailable, transcriptome sequencing through the use of differential expression studies is considered as a main way discovering novel genes in non-model organisms. Against this backdrop, to aid the basic understanding of withanolide biogenesis, transcriptome sequencing for *Withania* leaf (101L) and root (101R) synthesizing WS-3 and WS-1, respectively, has also been reported (Gupta et al., 2013b). Pyrosequencing results have yielded 834,068 and 721,755 reads assembled into 89,548 and 114,814 unique sequences from 101 L and 101 R. These are presumed to be involved in synthesis of tissue-specific withanolides. Annotations revealed all the genes involved in triterpenoid backbone biosynthesis that incorporated MVA and MEP pathways up to 24-methylene cholesterol, the apparent precursor for withanogenesis. Biosynthesis of 24-methylene cholesterol is followed by various secondary conversions including transfer of diverse moieties or oxidation/reduction reactions for structuring of tissue specific withanolides (Chaurasiya et al., 2012). Using gene Ontology and KEGG analyses, members of cytochrome P450, glycosyltransferase, and methyltransferase gene families with restricted presence or differential leaf and root expression have been reported. Quantification of reads for specific contig showed 305 contigs encoding CYP450s comprising of 12 and 36 unique CYPs for leaf and root tissues that may be responsible for the tissue specific difference in the activities counting withanolide biosynthesis. Unigene resource generated in this study also may be of immense value for interpretation of withanolide biosynthetic pathway and for search of tissue specific molecular mechanism fundamental for structuring definite withanolides (Gupta et al., 2013b).

## Comparative proteome analysis

Proteomic approach encompassing research centered on two dimensional electrophoresis (2-DE) and mass spectroscopy (MS) presents a new system for identifying known and unknown genes due to its ability to investigate hundreds of proteins simultaneously (Singh et al., 2015). This feasibility of proteomic analysis would make a considerable contribution in understanding the complex metabolic networks of withanolide biosynthesis in *W. somnifera* that could be a significant addition to the genomic knowledge resource. As a step forward, comprehensive 2-DE and MS analysis of *in vitro* grown adventitious roots and *in vivo* root samples of *W. somnifera* was conducted. The study showed a high similarity in protein spots of *in vitro* and *in vivo* root samples. Thus, suggesting that *in vitro* roots may have a analogous developmental route as that of *in vivo* roots though these are developed independent of shoot organs (Senthil et al., 2013). Cumulative proteome examination of leaf and seed tissues of *W. somnifera* differentiated the proteome on the basis of differential expression, count, and function of identified and characterized tissue-specific proteins. Relative examination of the two tissues further hinted that several proteins of common housekeeping pathways, while a few were tissue specific associated with definite metabolic complement (Dhar et al., 2012). Further, studies on low, abundant and poor soluble proteins would help in characterization of unknown pathway genes that are responsible for the production of withanolides.

Characterization of genes, their high throughput metabolic profiling, sequence resource and proteome information not only provides an insight into the withanolide biosynthetic pathway but also offers molecular wherewithals for biotechnological improvement of *W. somnifera*. However, in-depth knowledge about withanogenesis still remains elusive and all these results in totality could be useful to reveal various underlying signal transduction pathways to identify specific transcription factors in addition to uncharacterized downstream pathway genes. Further, such biosynthetic genes along with the transcription factors can become prospective targets for pathway engineering.

## Tissue culture approaches for withanolide production

Immense therapeutic value of *W. somnifera* attracts exploration of all possible approaches covering both recombinant DNA techniques and *in vitro* methods for obtaining chemotypes with desired enhanced chemoprofiles. Recombinant DNA or cell fusion techniques are viable alternatives, but are hampered by the lack of genetic and biochemical knowledge regarding the biosynthesis of secondary metabolites (Evans and Sharp, 1986). Thus, demanding immediate biotechnological advances to enhance the yield at a reduced time gap. On the other hand, in search of alternatives, *in vitro* techniques present a feasible option for the production of these therapeutically valuable compounds. Tissue culture techniques deliver unceasing, consistent, and renewable source of valued plant pharmaceuticals utilized for the large-scale culture of the plant cells necessary for extraction of secondary metabolites. Substantial work has been reported using different types of i*n vitro* strategies for *W. somnifera* with main emphasis on manipulation of plant growth regulator adjuvants and cultural conditions for withanolide accumulation. The prospect of application of *in vitro* methods to produce cell/organ/root cultures for enhanced withanolide production is reviewed below.

## Cell suspension culture

Various withanolides correspond to a very minor percentage of total withanolide content in the native plant. Investigation of condition adjusted cultures for resourceful *in vitro* biogeneration of such pharmacologically promising withanolides is important. Withanolide D, WS-1, WS-3, and WS-2 production have been described in organogenic cultures (Roja et al., 1991; Banerjee et al., 1994; Ray et al., 1996; Vitali et al., 1996; Ray and Jha, 1999; Furmanowa et al., 2001; Sangwan et al., 2007; Murthy et al., 2008). Several reports on accumulation of withanolide D and WS-3 in transformed roots/shooty teratomas cultures are also present, however WS-1 was reported to be absent in these cultures (Banerjee et al., 1994; Ray and Jha, 1999). Successful establishment of cell suspension cultures of *W. somnifera* for biogenesis of WS-1 has been reported and optimized for its enhanced accumulation.

Highest WS-1 content (1.27 mg g^−1^ DWB) was observed in suspension cultures supplemented with 2.0 mg L^−1^ 2,4-D, and 2.0 mg L^−1^ 2,4-D + 0.5 mg L^−1^ kinetin (KN). Thus, revealing that combination of 2,4-D with kinetin is the most suitable for enhanced WS-1 production. It has also been reported a combination of 1.0 ppm benzylaminopurine plus 0.5 ppm kinetin responsible for highest accumulation of WS-1 (14.3 mg per 100 g fresh weight and 238 mg per 100 g DWB, i.e., 0.24%) with the shoot cultures of *W. somnifera* (Table 2; Sangwan et al., 2007). Growth kinetics study of *W. somnifera* cell suspension cultures revealed maximum accumulation of biomass (11.02 g L^−1^ of DWB) and withanolide A (2.03 mg g^−1^ DWB) at the end of the fourth week. Therefore, making it clear that the biomass growth is closely concurrent with WS-1 amassing. Inoculum density of 10 g L^1^ was found to be the most suitable for maximum biomass (10.88 g L^−1^ DWB) and highest production of withanolide A (2.42 mg g^−1^ DWB). Among the different medias like Murashige and Skoog (MS), B5, NN, and N6, highest accumulation of WS-1 (2.39 mg g^−1^ DWB) was observed with full strength MS medium suspension culture supplemented with 3% sucrose with an initial medium pH of 6.0 (Table 2; Nagella and Murthy, 2010).

**Table 2:** ***In vitro* studies in *Withania somnifera* in relation to withanolides production**.

***In vitro* strategy used**	**Effect on withanolide levels**	**References**
**CELL SUSPENSION CULTURE**		
(a) Supplementation with 2.0 mg L^−1^ 2,4-D and 2.0 mg L^−1^ 2,4-D + 0.5 mg L^−1^ kinetin (b) Supplementation with 1.0 ppm benzylaminopurine plus 0.5 ppm kinetin (c) Murashige and Skoog medium suspension culture supplemented with 3% sucrose	(a) 1.27 mg g^−1^ DWB of WS-1 (b) 14.3 mg/100 gm fresh weight and 238 mg/100 gm DWB of WS-1 (c) 2.39 mg g^−1^ DWB of WS-1	https://www.jstage.jst.go.jp/article/cpb/55/9/55_9_1371/_article http://www.sciencedirect.com/science/article/pii/S0960852410005559
***IN VITRO* SHOOT CULTURE**		
(a) Supplementation with 1.00 ppm benzylaminopurine and 0.50 ppm kinetin (b) Elicitation with salicylic acid at 100 μM in combination with 0.6 mg L^−1^ 6-benzyladenine and 20 mg L^−1^ spermidine	(a) 14.3 mg per 100 g FWB and 238 mg per 100 g DWB of WS-1 (b) 1.14− to 1.18-fold higher withanolide production	http://jlc.jst.go.jp/JST.JSTAGE/cpb/55.1371?from=Google http://link.springer.com/article/10.1007/s11738-012-1112-x#page-1
**ROOT CULTURE**		
(a) MS-based liquid medium supplemented with 40 g/L sucrose for hairy roots (b) Adventitious root cultures elicited with 150 μM salicylic acid	(a) 2.7-fold higher WS-1 (b) 64.65 mg g^−1^ DWB of WS-1 (48-fold), 33.74 mg g^−1^ DWB of withanolide B (29-fold), 17.47 mg g^−1^ DWB WS-3 (20-fold), 42.88 mg g^−1^ DWB WS-2 (37-fold), 5.34 mg g^−1^ DWB 12-deoxy withastramonolide (nine-fold), 7.23 mg g^−1^ DWB withanoside V (seven-fold), and 9.45 mg g^−1^ DWB withanoside IV	http://onlinelibrary.wiley.com/doi/10.1111/j.1744-7909.2008.00680.x/full http://link.springer.com/article/10.1007/s12010-012-9809-2#page-1
**SOMACLONAL VARIANTS**		
Somaclonal variants	Somaclonal variant regenerated with 0.516% (DWB) of 12-deoxywithastramonolide	http://link.springer.com/article/10.1007/s11627-012-9458-8

Investigations have also been carried on the biotransformation capacity of cell suspension cultures generated from *W. somnifera* leaf using WS-1, WS-3, and WS-2 as precursor substrates. Interestingly, there was a noticeable inter-conversion of WS-1 to WS-2, and vice versa involving substitution of 20-OH group to 17-OH in WS-1 (Sabir et al., 2011). It displays the potential of suspension cultures of *W. somnifera* for the production of withanolides with multifactorial modulations.

## *In vitro* shoot culture

Root specific production of WS-1 makes root cultures, particularly hairy roots, and its bioreactor based upscaling the foremost way for its *in vitro* production. However, presence of WS-1 has not been detected in *Agrobacterium rhizogene*-transformed hairy roots of *W. somnifera* (Banerjee et al., 1994; Ray and Jha, 1999). Withanolides detected in these hairy root cultures have been reported to be predominantly produced by the aerial parts. Therefore, WS-1 biogenesis was explored in *W. somnifera* shoot cultures that are the tissue culture complements of the aerial parts of the native plant. Shoot cultures were initiated using explants from the two experimental lines of *W. somnifera* (Ashwagandha)-*RS Selection-1* (*RS-Sel-1*) and *RS Selection-2* (*RS-Sel-2*) on MS medium with different plant growth regulators. *RS-Sel-1* raised shoot culture supplemented with benzylaminopurine (BAP) 1.00 ppm and KN 0.50 ppm showed the highest concentration of WS-1 (14.3 mg per 100 g fresh weight and 238 mg per 100 g dry weight, i.e., 0.24%) in the green shoots (Table 2). Investigative quantities of green shoot cultures (0.24% DWB) was more as compared to the isolation yields of dried roots of field-grown plants. Solid mass/shooty teratoma of *RS-Sel-1* raised shoot culture also displayed the highest concentration of WS-1 production (3.7 mg per 100 g fresh weight; 46.2 mg per 100 g dry weight) with BAP 1.00 ppm and kinetin 0.50 ppm. Comparatively, *RS-Sel-1* proved to have superior biogenesis/accumulation of WS-1 than *RS-Sel-2*. Radioactivity fed shoot cultures as well led to isolation of almost pure radiolabeled WS-1 and pointed toward *de novo* biosynthesis of WS-1 in the *in vitro* shoot cultures (Sangwan et al., 2007).

The effect of hormones, culture conditions and elicitations on the production of withanolides in multiple shoot cultures of *W. somnifera* has also been reported. Elicitation with salicylic acid at 100 μM in combination with 0.6 mg L^−1^ 6-benzyladenine and 20 mg L^−1^ spermidine for 4 h at the fourth week in 20 ml liquid medium reported 1.14− to 1.18-fold higher withanolide production in comparison to the elicitation treatment with MJ at 100 μM after 5 weeks of culture (Table 2; Sivanandhan et al., 2013). Hence, confirming *in vitro* shoot culture biosystems as an alternative amenable to fine-tuning for harvesting therapeutically valuable WS-1 in comparison to field grown plants. Shoot cultures also represent a suitable system for functional genomic studies of withanolides.

## Root culture

Hairy (transformed) roots mediated with *A. rhizogenes* holds immense potential for studies on secondary metabolite biosynthesis as rapid growth and extensive branching with genetic stability is their characteristic feature. They also exhibit capability of synthesizing root specific secondary metabolites (Giri and Narasu, 2000; Hu and Du, 2006). Consequently, hairy roots in numerous aromatic and medicinal plants for the production of significant secondary compounds have been induced (Le Flem-Bonhomme et al., 2004; Zhao et al., 2004; Santos et al., 2005). Murthy et al. (2008) reported transformation of *W. sominifera* with *A. rhizogenes* strain R1601 and obtained transformed hairy roots from cotyledons and leaf explants. Four clones of hairy roots differing in morphology were established. MS-based liquid medium supplemented with 40 g/L sucrose proved to be optimum for biomass building. WS-1 content was found to be 2.7-fold higher in transformed roots (line 3) in comparison to non-transformed roots (Table 2; Murthy et al., 2008).

Leaf derived callus of *W. somnifera* has also been used for development of adventitious root cultures in MS half-strength medium supplemented with 0.5 mg L^−1^ indole-3-butyric acid (IBA) and 0.1 mg L^−1^ indole-3-acetic acid (IAA) with 2% sucrose. These adventitious root cultures were further elicited with MJ and SA autonomously to investigate the improvement in the productivity of withanolides. Root biomass (11.70 g FWB) on 30-day-old adventitious root cultures treated with 150 μM SA for 4 h resulted in the production of 64.65 mg g^−1^ DWB WS-1 (48-fold), 33.74 mg g^−1^ DWB withanolide B (29-fold), 17.47 mg g^−1^ DWB WS-3 (20-fold), 42.88 mg g^−1^ DWB WS-2 (37-fold), 5.34 mg g^−1^ DWB 12-deoxy withastramonolide (nine-fold), 7.23 mg g^−1^ DWB withanoside V (seven-fold), and 9.45 mg g^−1^ DWB withanoside IV (nine-fold) following elicitation of 10 days (40th day of culture) in comparison to untreated cultures (Table 2). Withanolide production was found to be dependent on biomass, culture age of the adventitious roots, elicitation concentration and time period involved (Sivanandhan et al., 2012). Thus, highlighting the considerable potential of transformed roots and adventitious roots of *W. somnifera* with further *s*cale-up in bioreactors.

## Somaclonal variants

Somaclonal variation can be either genetic or epigenetic in origin (Larkin and Scowcroft, 1981; Lee and Phillips, 1988). The occurrence of somaclonal variation is often associated with activation of transposable elements (Skirvin et al., 1994), point mutation, chromosomal rearrangement, recombination, DNA methylation, and altered sequence copy number. Somaclonal variation is influenced by explant type, culture medium, genotype, and the age of the donor plant, among other factors (Skirvin et al., 1994). It has been suggested that the frequency of somaclonal variation from cell culture is much higher than from field-grown plants because of a, higher rate of mutagenesis (Ahloowalia, 1986). Many plants regenerated via indirect organogenesis have shown somaclonal variation for a wide array of characteristics and this variation have been used to develop new varieties in some species, like tomato, sorghum, sugarcane, and chrysanthemum (Compton and Veilleux, 1991; Duncan et al., 1995; Jalaja et al., 2006; Miñano et al., 2009). Somaclonal variation could unleash the natural variability for withanolide production and accumulation, and could be exploited by breeders to develop *W. somnifera* varieties attracting commercial interest.

Rana et al. (2012) investigated and validated the applicability of an *in vitro* strategy to induce somaclonal variation in *W. somnifera* which manifested in the form of enhanced levels of 12-deoxywithastramonolide (WS-12D). Variations were examined in 54 regenerated plants obtained through indirect organogenesis from leaf explants. WS-R-1 somaclone displayed considerably elevated levels of WS-12D; 0.516% DWB in comparison to the explant donor mother plant (0.002% DWB). Somaclonal variations were investigated at cytological level, by investigating meiosis and mitosis in comparison to number of chromosome and structural organization. Chromosome phenotypes, somatic chromosome count, or meiotic behavior showed no alterations. Further, several genetic polymorphisms between explant donor mother plant and WS-12D over-producing somaclone was examined by random amplification of polymorphic DNA (RAPD) study. WS-R-1 somaclone was evaluated for 2 years to confirm genetic and chemical stability. This study supports the feasibility of an *in vitro* strategy for chemotypic variability induction to develop high-yielding clones considering the molecular instability displayed by *W. somnifera*. It also widens the genetic resource base for manipulative hybridization for quantitative chemotypic novelty in *W. somnifera* (Rana et al., 2012).

## Future prospects

*W. somnifera* has enjoyed a long and important history in traditional medicine system wherein withanolides are attributed with significant remedying properties. Nevertheless, withanolide biosynthesis is still in its infancy with regard to being understood in entirety that enormously hampers the exploitation of its full biotechnological potential. Though, investigations at molecular and *in vitro* levels have begun, but we are still a long way from understanding how diverse withanolides are synthesized and regulated in *W. somnifera*. However, the gene elucidation data, omics resource and *in vitro* study inferences generated so far offers significant promise for enormous increase in correct annotation, functional characterization of enzymes and for comprehending the assorted interactions amongst sophisticated biosynthetic and regulatory mechanisms crucial for successful implementation of withanolide metabolic engineering strategies. Furthermore, advancing metabolic engineering technologies for transgenics, precursor feeding, gene overexpression and inhibition and mutant selection in *W. somnifera* still awaits investigation. There is much to be learned about the chemical ecology of withanolides to answer an important question about their evolution in the form of sophisticated and diverse structures and types. Though, anticipation about withanolides acting as growth regulators owing to their partial coinciding biosynthetic route with brassinosteroids do exist, but it further demands in-depth examination to build a framework for elaborate pathway modulation strategies.

## Author contributions

SL, RV conceived and designed the review. ND and SL wrote the manuscript. ND, S Razdan, S Rana, WB, SL, and RV have contributed in the original studies published earlier vis-à-vis *W. somnifera*. ND, S Razdan, S Rana, and WB have also collated the up-to-date literature. All authors read and approved the final manuscript.

### Conflict of interest statement

The authors declare that the research was conducted in the absence of any commercial or financial relationships that could be construed as a potential conflict of interest.
